# MiR-145-5p suppresses the proliferation, migration and invasion of gastric cancer epithelial cells via the *ANGPT2/*NOD_LIKE_RECEPTOR axis

**DOI:** 10.1186/s12935-020-01483-6

**Published:** 2020-08-28

**Authors:** Kai Zhou, Binbin Song, Ming Wei, Jubo Fang, Yufen Xu

**Affiliations:** 1 Department of General Surgery, The Dongda Hospital, Shanxian, Heze, 274000 China; 2 Department of Oncology, The First Hospital of Jiaxing, Affiliated Hospital of Jiaxing University, 1882# Zhonghuan South Road, Jiaxing, 314000 China

**Keywords:** miR-145-5p, *ANGPT2*, NOD_LIKE_RECEPTOR, Gastric cancer, Proliferation, Migration and invasion

## Abstract

**Objective:**

This study aimed to investigate the relationship among miR-145-5p, *ANGPT2* and the NOD_LIKE_RECEPTOR pathway, thereby revealing the molecular mechanism of these three factors underlying the proliferation, migration and invasion of gastric cancer (GC) epithelial cells.

**Methods:**

qRT-PCR was carried out to detect the expression of miR-145-5p and *ANGPT2* mRNA. Western blot was performed to test the protein levels of ANGPT2 as well as NOD1, NOD2 and NF-κB in the NOD_LIKE_RECEPTOR pathway. The targeting relationship between miR-145-5p and *ANGPT2* was verified via a dual-luciferase reporter gene assay. The proliferation, migration and invasion of GC cells were detected through MTT and Transwell assays, respectively.

**Results:**

The expression of miR-145-5p was significantly down-regulated in GC cells, while that of *ANGPT2* was notably up-regulated. MiR-145-5p directly bound with the 3′-UTR of *ANGPT2* mRNA, thereby targeting *ANGPT2* after transcription. Overexpression of miR-145-5p inhibited the proliferation, migration and invasion of GC cells by suppressing *ANGPT2*. Moreover, low expression of *ANGPT2* affected the protein levels of NOD1, NOD2 and NF-κB in the NOD_LIKE_RECEPTOR pathway, thus weakening the abilities of cell proliferation, migration and invasion.

**Conclusions:**

MiR-145-5p plays an important role in GC epithelial cells, and it can affect cell proliferation, migration and invasion of GC cells by targeting *ANGPT2* and regulating the NOD_LIKE_RECEPTOR pathway. Overall, our study further elucidates the molecular mechanism underlying the malignant progression of GC.

## Background

Gastric cancer (GC) is one of the four most common malignancies worldwide with a relatively high rate of morbidity and mortality [1]. Because of unobvious symptoms in the early stage of GC, most patients are diagnosed in the advanced stage, and surgical resection is no more effective, leading to a low cure rate and poor overall prognosis [2]. In addition, the incidence of GC in young people has gradually increased [3]. Although great progress has been achieved in the diagnosis and treatment of GC in recent years, the survival rate of this disease in most countries still remains 25–30% [4]. Therefore, investigating the molecular mechanism underlying GC initiation and progression is of great importance for exploring targeted therapeutic approaches towards GC .

MicroRNAs (miRNAs), a type of small endogenous RNA, can negatively regulate gene expression in the post-transcriptional level [5–7], and most of them can pair with the complementary sites within their target mRNAs [8]. Previous studies suggested that miRNAs are involved in the proliferation, migration and invasion of cancer cells [9, 10]. For instance, miR-137 plays an anti-tumor role in astrocytoma cells through targeting *RASGRF1* [10]. In esophageal squamous carcinoma, miR-125b targets *BMF*, thereby inhibiting cell proliferation and inducing apoptosis [11]. Furthermore, miR-183-5p and miR-486-5p are found to be significantly correlated with TNM staging in GC patients, while miR-30c-2-3p and miR-133a-3p are associated with the degree of tumor differentiation and lymph node metastasis [12].

Angiogenesis is the formation of new blood vessels from the pre-existing ones. These new blood vessels can be used to form a new vascular network in tumor, thereby promoting a series of cell activities, such as proliferation, migration, invasion and adhesion. Therefore, angiogenesis is crucial during the occurrence and development of various malignant tumors [13–15]. More evidence has revealed that *Angiopoietin-2* (*ANGPT2*, *Ang2*) can function on angiogenesis and directly stimulate the proliferation of cancer cells [16]. In addition, as an antagonist of *ANGPT1*, *ANGPT2* can suppress the autophosphorylation of *Tie2* and break the stabilization of blood vessels. In the presence of vascular endothelial growth factors (VEGFs), vessels that are damaged might be repaired with the occurrence of angiogenesis. In other words, *ANGPT2* can cooperate with VEGFs to induce angiogenesis, whereas *ANGPT1* can offset VEGFs-induced angiogenesis in vivo [17, 18]. As the level of *ANGPT2* is up-regulated in plasma in cancer patients and associated with poor prognosis [19–21], *ANGPT2* can be used as a biomarker for cancer treatment.

NOD-like receptor (NLR) is a type of pattern recognition receptor (PRR) that plays a vital role in immunoregulation [22]. It has been reported that NLR-mediated inflammation is associated with several tumor activities, such as tumorigenesis, progression, metastasis and survival [23]. Additionally, NLR signaling pathways have a significant role in numerous human diseases, including bacterial infections, autoimmune and inflammatory disorders, and cancers [22].

Studies have suggested that miRNAs can be involved in the regulation of angiogenesis via affecting the angiogenesis factors in endothelial cells [24]. For example, miR-126 [25] and miR-210 [26] can facilitate angiogenesis, while miR-221 and miR-222 [27] play a negative role. In the present study, we investigated the regulatory relationship between miR-145-5p and *ANGPT2*, thereby revealing the effect of these two factors on the cell proliferation, migration and invasion of GC.

## Materials and methods

### Cell culture

Human GC cell lines BGC-823 (BNCC100086), MGC-803 (BNCC340396), SGC-7901 (BNCC100114), HGC-27 (BNCC100097), AGS (BNCC102154) and normal gastric cell line GES-1 (BNCC342032) were purchased from BeNa Culture Collection (Beijing, China). All cells were cultured in the RPMI-1640 medium (Gibco, Thermo Fisher Scientific, Inc., Waltham, MA, USA) supplemented with 10% fetal bovine serum (FBS; Hyclone; GE Healthcare Life Sciences, Logan, UT, USA) as well as 100 U/mL streptomycin (Gibco, Thermo Fisher Scientific, Inc.) and 100 U/mL penicillin (Gibco, Thermo Fisher Scientific, Inc.) under the environment of 5% CO_2_ at 37 ℃. The medium was replaced on a regular basis.

### Cell transfection

MiR-145-5p mimics and NC mimics were purchased from Ribo Bio (Guangzhou, People’s Republic of China). Si-ANGPT2 and si-NC were ordered from GenePharma (Shanghai, China). Lentiviral expression vector pLVX-IRES-neo (Clontech, USA) was used to construct *ANGPT2* overexpression vector (oe-ANGPT2), which was then used to infect GC cells with the empty pLVX-IRES-neo vector as control. For preparation, all cells were cultured in complete medium for at least 24 h, and then rinsed by phosphate buffered saline (PBS, pH7.4). Transfection was carried out using Lipofectamine2000 (Thermo Fisher Scientific, Inc.), and cells were cultured in corresponding mediums with 5% CO_2_ at 37 ℃. Blank group was taken as the control only with the transfection reagent.

### qRT-PCR

Total RNA of GC cells was extracted by Trizol kits (Thermo Fisher Scientific), and then taken for reverse transcription using the cDNA synthesis kits (Thermo Fisher Scientific) for the access of cDNA.

qRT-PCR was carried out under the thermal cycling conditions using the miScript SYBR Green PCR Kits (Qiagen, Hilden, Germany): pre-degeneration at 95 ℃ for 10 min, 40 cycles of 95 ℃ for 15 s, 60 ℃ for 30 s followed by 72 ℃ for 30 s. *U6* and *GAPDH* were taken as the internal references for normalization of the expression levels of miR-145-5p and *ANGPT2* mRNA, respectively. Primers used were all from GeneCopoeia (Guangzhou, China), and sequences are listed in Table 1.

**Table 1:** Primer sequence

Gene	Primer sequence
miR-145-5p [28]	F: 5′-GTCCAGTTTTCCCAGGAATC-3′
	R: 5′-AGAACAGTATTTCCAGGAAT-3′
U6	F: 5′-CTCGCTTCGGCAGCACA-3′
	R: 5′-AACGCTTCACGAATTTGCGT-3′
ANGPT2 [29]	F: 5′-AGATTTTGGACCAGACCAGTGA-3′
	R: 5′-GGATGATGTGCTTGTCTTCCAT-3′
GAPDH	F: 5′-GGAGCGAGATCCCTCCAAAAT-3′
	R: 5′-GGCTGTTGTCATACTTCTCATGG-3′

Relative expression levels of the target mRNA and miRNA in the control and experimental groups were compared with the 2^−ΔΔCt^ value. The experiment was repeated three times.

### Western blot

After 48 h of incubation, cells were washed by cold PBS three times and lysed on ice with whole cell lysate for 10 min. Proteins contained were quantitated by BCA protein assay kit (Thermo Fisher Scientific, Waltham, MA, USA). A measure of 30 µg of total proteins were then separated by polyacrylamide gel electrophoresis (PAGE) at a constant voltage of 80 V for 35 min followed by 120 V for 45 min, and sequentially transferred onto the polyvinylidene fluoride (PVDF) membranes (Amersham, USA) after electrophoresis. The membranes were blocked with 5% skim milk at room temperature for 1 h. Afterwards, the membranes were incubated overnight at 4 ℃ with primary rabbit polyclonal antibodies containing ANGPT2 (ab155106, 1:1000, abcam, Cambridge, UK), NOD1 (ab217798, 1:1000, abcam), NOD2 (ab36836, 1:1000, abcam), NF-κB (ab194729, 1:1000, abcam) and GAPDH (ab9485, 1:2500, abcam). Then, the membranes were incubated with secondary antibody, horse radish peroxidase-labeled goat anti-rabbit IgG H&L (HRP) (ab6721, 1:2000, abcam) for 1 h. Protein bands were visualized under the optical luminescence instrument (GE, USA), and were quantified by gray scale scanning using the Image Pro Plus 6.0 software (Media Cybernetics, USA).

### MTT assay

MTT method (Sigma, Shanghai) was applied for proliferation detection of GC cells. Cells were seeded into 96-well plates at a density of 2 × 10^3^ cells/well. After 24 h, 48 and 72 h, respectively, 20 µL of MTT (0.5 mg/mL; Sigma-Aldrich, St. Louis, MO, USA) was added into each well for incubation at 37 ℃ for 4 h. The precipitate was solubilized in 150 µL of RPMI-1640. The OD values at 570 nm were recorded. The experiment was repeated three times.

### Transwell

#### Migration assay

Cells in the logarithmic phase were firstly starved for 24 h. On the following day, cells were digested, centrifuged and resuspended to a concentration of 2 × 10^5^ cells/mL. 0.2 mL of cell suspension was added into the top chambers, and 700 µL pre-cooled RPMI-1640 medium complemented with 10% FBS was added into the bottom chambers. After incubation for 24 h at 37 ℃ with 5% CO_2_, the non-migrated cells in the top chambers were wiped off with a cotton swab, and the migrated cells were fixed with methanol for 30 min and stained with 0.1% crystal violet for 20 min. Then, cells were rinsed, dried and observed under an inverted microscope. Five fields of view were randomly selected for cell count.

#### Invasion assay

Transwell invasion assays were performed using 24-well Transwell chambers (8 µm in aperture, BD Biosciences). GC cells (2 × 10^4^) were added into the top chambers coated by Matrigel matrix (Corning, NY), and RPMI-1640 medium containing 10% FBS was added to the bottom chambers. After incubation at 37 ℃ for 24 h, cotton swabs were used to wipe off the cells still in the top chambers, and the invaded cells were stained with crystal violet. Cells were counted in 5 randomly selected fields.

### Dual-luciferase reporter assay

GC cells were seeded into 24-well plates in a density of 6 × 10^5^ cells/well for 24 h of incubation. Wild type (WT) and mutant (MUT) *ANGPT2* 3′-UTR were respectively cloned into pmiRGLO (Promega, Madison, WI, USA) vectors for construction of luciferase reporter vectors WT- ANGPT2 and MUT-ANGPT2. The two vectors were respectively co-transfected with miR-145-5p mimics/NC mimics into GC cells, with the Renilla luciferase expression vector pRL-TK (TaKaRa, Dalian, China) as the internal reference. After transfection, cells were cultured in RPMI-1640 medium containing 10% FBS for 48 h. Dual-luciferase reporter gene assay system (Promega, Madison, WI, USA) was used for luciferase activity examination. The experiment was repeated three times.

### Statistical analysis

All data were processed by SPSS 21.0 statistical software. Measurement data were presented in the form of mean ± standard deviation (SD). Comparison between two groups was performed by *t* test, and comparison among multiple groups was analyzed by one-way ANOVA. *p* < 0.05 was considered statistically significant.

## Results

### MiR-145-5p is poorly expressed in GC, while *ANGPT2* is highly expressed

**Fig. 1:**
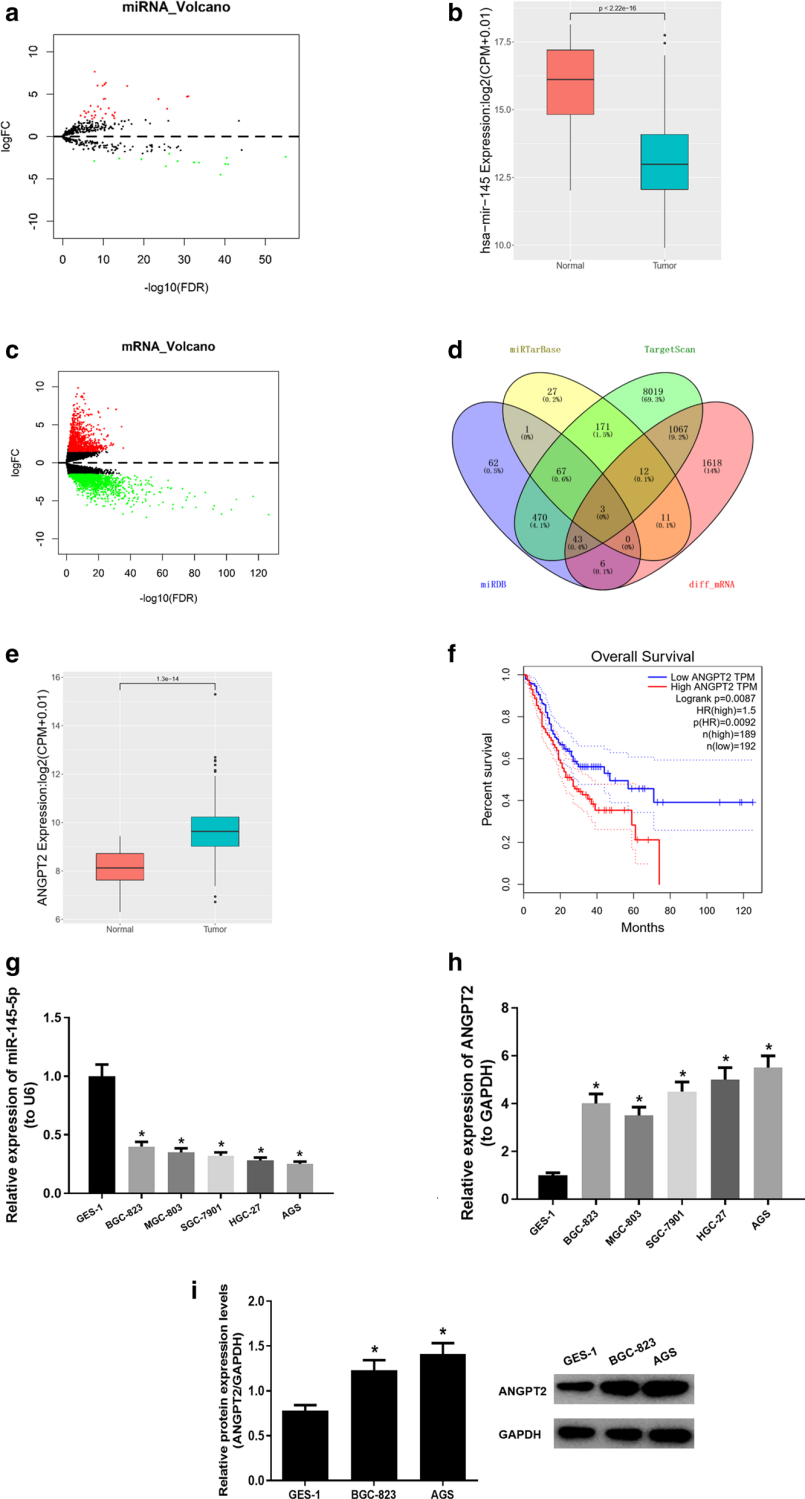
MiR-145-5p is poorly expressed in GC, while *ANGPT2 *is highly expressed. **a** Volcano plot shows the 46 differentially expressed miRNAs in TCGA-STAD dataset; **b** Differential expression of miR-145-5p in normal and tumor groups in TCGA-STAD samples; **c** Volcano plot shows the 2760 differentially expressed mRNAs in TCGA-STAD dataset; **d** Venn diagram is plotted to find the overlapping mRNAs from the differentially expressed mRNAs in TCGA-STAD dataset and the target genes of miR-145-5p predicted in three bioinformatics databases; **e** Differential expression of *ANGPT2* in normal and tumor groups in TCGA-STAD samples; **f** Overall survival curves of patients in high (red) and low (blue) *ANGPT2* expression groups; **g**, **h** The expression of miR-145-5p and *ANGPT2* mRNA in 5 GC cell lines BGC-823, MGC-803, SGC-7901, HGC-27, AGS and one normal gastric cell line GES-1 is assessed by qRT-PCR; **i** The protein expression of ANGPT2 in BGC-823 and AGS cell lines; *p < 0.05

Expression profiles of STAD-associated miRNAs were accessed from TCGA database, including 45 normal samples and 444 GC tissue samples. “edgeR” package of R language was used to perform differential analysis with the normal samples as control. |logFC|>2 and adj. p value < 0.01 were set as the threshold. As shown in Fig. 1a and 46 differentially expressed miRNAs were obtained, among which miR-145 was found to be stably expressed in both the normal and cancer samples, but the expression showed a remarkable difference and the FDR value was statistically significant (Fig. 1b). According to the starBase database (http://starbase.sysu.edu.cn/index.php), miR-145-5p is considered the most probable mature form of miR-145.

It has been reported that miR-145-5p is closely related to tumorigenesis and progression [30, 31]. In order to investigate the downstream regulatory mechanism of miR-145-5p, we firstly screened differentially expressed mRNAs in TCGA-STAD dataset via differential analysis (Fig. 1c). Afterwards, TargetScan (http://www.targetscan.org/vert_71/), miRDB (http://www.mirdb.org/microDB/policy.html) and miRTarBase (http://mirtarbase.mbc.nctu.edu.tw/php/index.php) three databases were used to predict potential target genes of miR-145-5p. Venn diagram in Fig. 1d was drawn to find the overlapping mRNAs between the target genes predicted and the differentially expressed mRNAs obtained before. Eventually, 3 potential target genes of miR-145-5p were achieved, among which *ANGPT2* was significantly up-regulated in tumor samples (Fig. 1e). Moreover, 489 samples in TCGA-STAD dataset were grouped into high and low expression groups with the median expression of *ANGPT2* as the critical value. Further survival analysis revealed that *ANGPT2* expression showed a remarkable effect on prognosis of GC patients, and patients with high expression of *ANGPT2* had poor prognosis (Fig. 1f).

To investigate the role of miR-145-5p and *ANGPT2* in GC progression, expression levels of the two genes were detected in 5 GC cell lines BGC-823, MGC-803, SGC-7901, HGC-27, AGS and one normal gastric cell line GES-1. As shown in Fig. 1g, h, results of qRT-PCR suggested that the expression of miR-145-5p was significantly down-regulated in 5 GC cell lines by comparison with that in normal cell line GES-1, while *ANGPT2* had the opposite effect. For preciseness, BGC-823 and AGS with most significant differential expression of miR-145-5p or *ANGPT2* were selected for subsequent experiments. Western blot was performed to assess the protein levels of *ANGPT2* in these two cell lines (Fig. 1i), and the result obtained was consistent with that by qRT-PCR.

### MiR-145-5p inhibits the proliferation, migration and invasion of GC cells

**Fig. 2:**
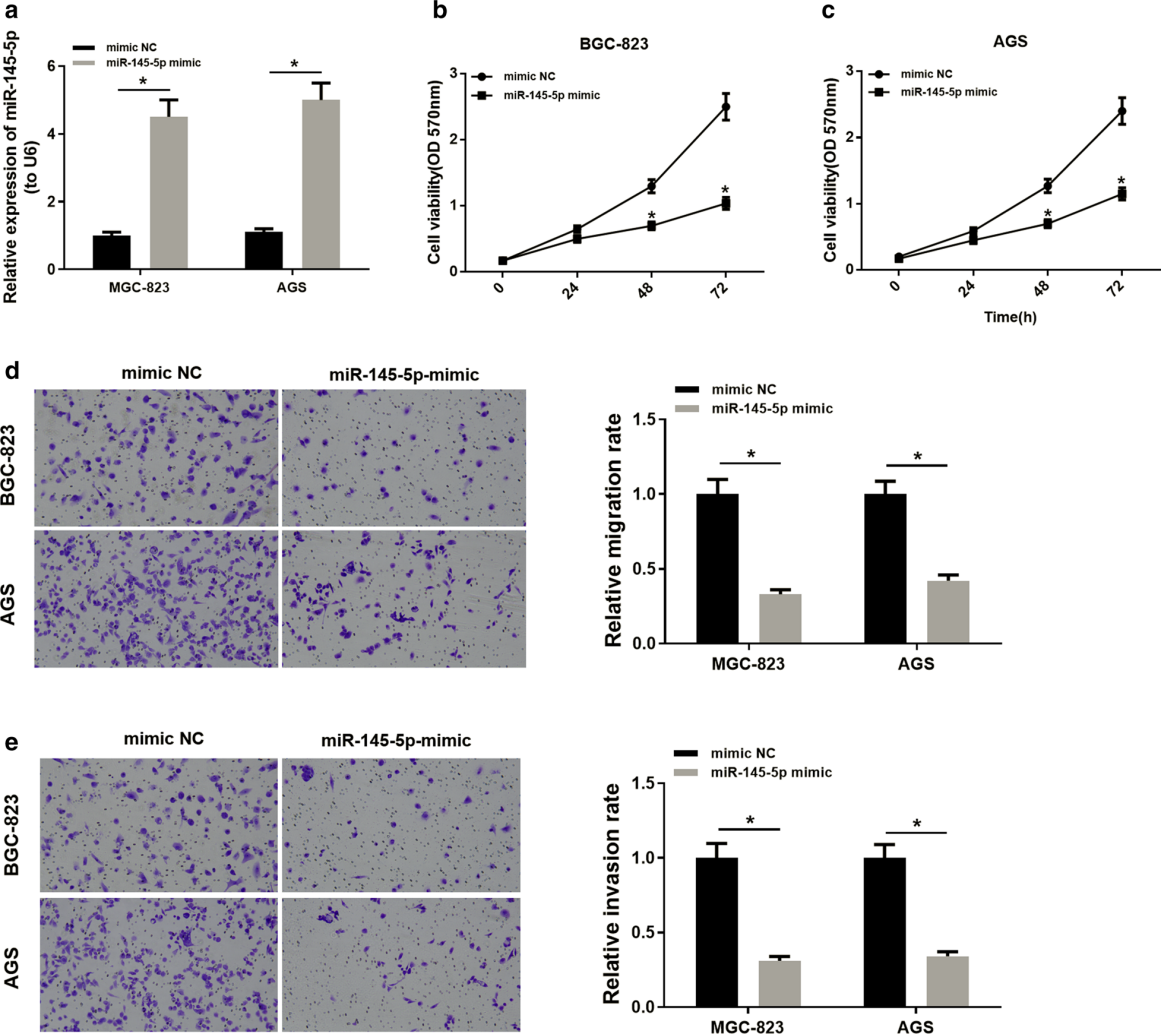
MiR-145-5p inhibits the proliferation, migration and invasion of GC cells. MiR-145-5p mimic and NC mimic were respectively transfected into BGC-823 and AGS cells. **a** The expression of miR-145-5p in two groups is detected by qRT-PCR; **b**, **c** Cell proliferation of BGC-823 and AGS cells in two groups is evaluated by MTT assay; **d** Cell migration of BGC-823 and AGS cells in two groups is evaluated by Transwell migration assay; **e** Cell invasion of BGC-823 and AGS cells in two groups is evaluated by Transwell invasion assay; *p < 0.05

NC mimic and miR-145-5p mimic were transfected into GC cell lines BGC-823 and AGS, respectively. Results of qRT-PCR (Fig. 2a) showed that the expression of miR-145-5p was significantly up-regulated in the miR-145-5p mimic group. MTT assay suggested that cells transfected with miR-145-5p mimic had much lower viability at 48 and 72 h (Fig. 2b, c). In addition, Transwell assays revealed that overexpression of miR-145-5p played an inhibitory role in cell migration (Fig. 2d) and invasion (Fig. 2e). All these data elucidated that miR-145-5p overexpression could inhibit the proliferation, migration and invasion of GC cells and in turn functioned in tumorigenesis and progression.

### MiR-145-5p targets *ANGPT2*

**Fig. 3:**
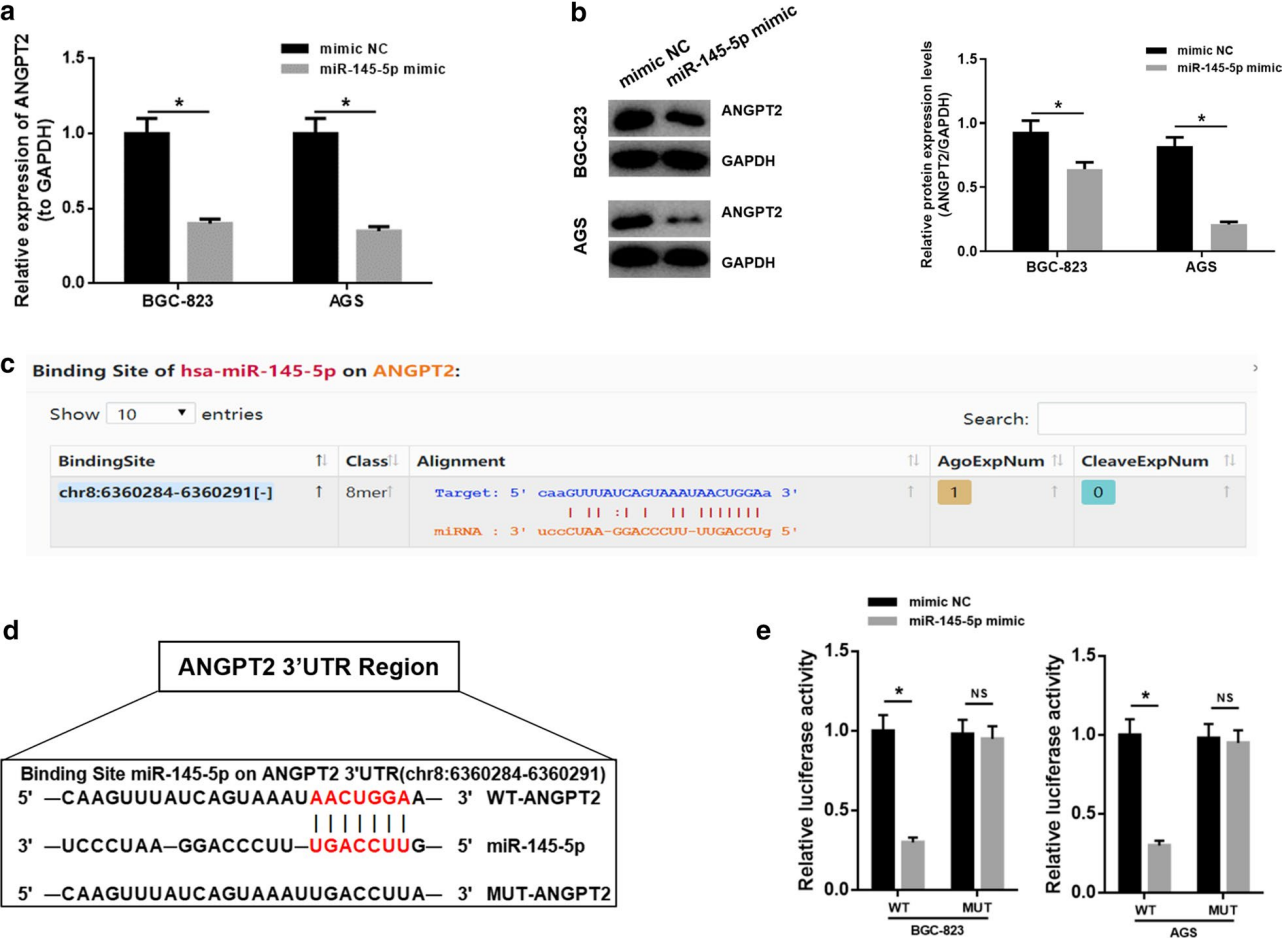
*ANGPT2 *is a target of miR-145-5p. **a** The expression levels of *ANGPT2* mRNA in the miR-145-5p mimic and NC mimic groups are tested by qRT-PCR; **b** The protein expression levels of ANGPT2 in BGC-823 and AGS cells are examined by western blot; **c** Binding sites of miR-145-5p on *ANGPT2* 3′-UTR are predicted by a bioinformatics database; **d** Sequences of WT-ANGPT2 and MUT-ANGPT2; **e** Relative luciferase activity in BGC-823 and AGS cells transfected with either WT-ANGPT2 or MUT-ANGPT2 is assayed by dual-luciferase assay; *p < 0.05

As described by bioinformatics analysis, the expression of miR-145-5p was found to be down-regulated in GC cells, while *ANGPT2* was up-regulated. In order to verify the regulatory relationship between miR-145-5p and *ANGPT2*, qRT-PCR and western blot were carried out. As shown in Fig. 3a, b, the mRNA and protein levels of ANGPT2 in the miR-145-5p mimic group were both remarkably decreased by comparison with those in the NC mimic group. Besides, TargetScan website was applied and it was found that there were potential targeted binding sites of miR-145-5p on *ANGPT2* 3′-UTR region (Fig. 3c). For further verification, luciferase reporter vectors WT-ANGPT2 and MUT-ANGPT2 were constructed (Fig. 3d). As shown in Fig. 3e, in the presence of miR-145-5p overexpression, luciferase activity in the cells transfected with WT-ANGPT2 presented a downward trend, while that with MUT-ANGPT2 showed no significant difference. All above findings indicated that miR-145-5p could directly target to *ANGPT2*.

### *ANGPT2 *inhibits the proliferation, migration and invasion of GC cells by regulating the NOD_LIKE_RECEPTOR pathway

**Fig. 4:**
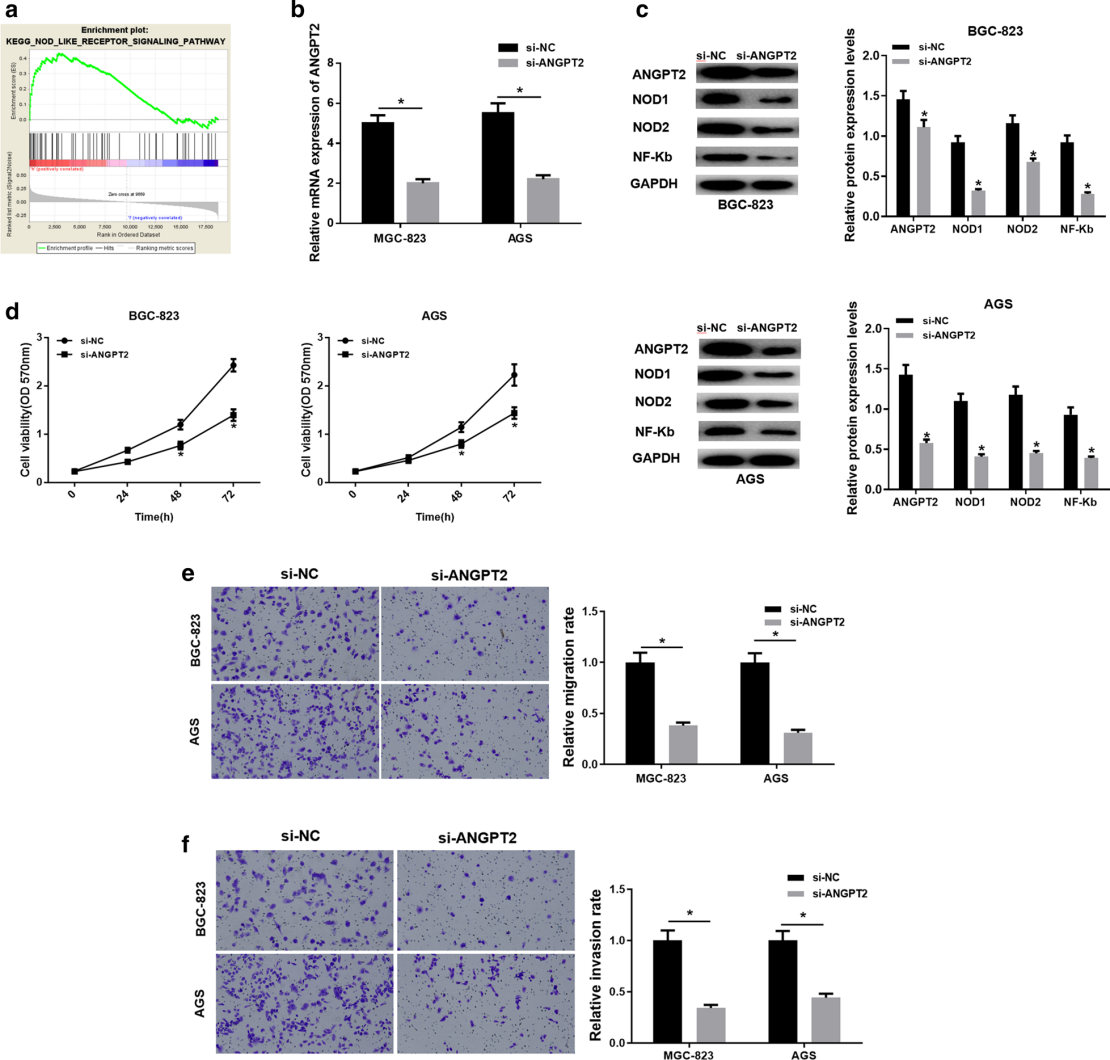
*ANGPT2 *can regulate the NOD_LIKE_RECEPTOR pathway. **a** Enrichment analysis shows the correlation between high *ANGPT2* expression and the NOD_LIKE_RECEPTOR signaling pathway; **b** The expression levels of *ANGPT2* mRNA in si-NC and si-ANGPT2 groups in BGC-823 and AGS cells are determined by qRT-PCR; **c** Protein levels of ANGPT2, NOD1, NOD2 and NF-κB in BGC-823 and AGS cells in two groups are tested by western blot; **d** Cell migration of BGC-823 and AGS cells in two groups is evaluated by Transwell migration assay; **e** Cell migration of BGC-823 and AGS cells in two groups is evaluated by Transwell migration assay; **f** Cell invasion of BGC-823 and AGS cells in two groups is evaluated by Transwell invasion assay; *p < 0.05

GSEA enrichment analysis was conducted and the result shown in Fig. 4a suggested that *ANGPT2* was mostly enriched in the NOD_LIKE_RECEPTOR pathway and NOD1, NOD2 and NF-κB are the key proteins involved in. Therefore, these three key proteins could be used to verify the regulatory effect of *ANGPT2* on the NOD_LIKE_RECEPTOR pathway. Results of qRT-PCR in Fig. 4b showed that the expression of *ANGPT2* was significantly decreased in the si-ANGPT2 group. Meanwhile, western blot indicated that the protein levels of NOD1, NOD2 and NF-κB were correspondingly decreased in the cells with low ANGPT2 expression (Fig. 4c). Findings above suggested that *ANGPT2* could affect the NOD_LIKE_RECEPTOR pathway. In addition, MTT and Transwell assays were performed to verify the effect of *ANGPT2* on cancer cell proliferation, migration and invasion. As shown in Fig. 4d, cancer cell proliferation was significantly inhibited in the si-ANGPT2 group. Similarly, the migration and invasion of BGC-823 and AGS cells were suppressed as well when *ANGPT2* expression was decreased (Fig. 4e, f). Overall, our study confirmed that low expression of *ANGPT2* could reduce the proliferation, migration and invasion of GC cells, and suppress its downstream NOD_LIKE_RECEPTOR pathway.

### MiR-145-5p suppresses the *ANGPT2*/NOD_LIKE_RECEPTOR axis to affect GC progression

**Fig. 5:**
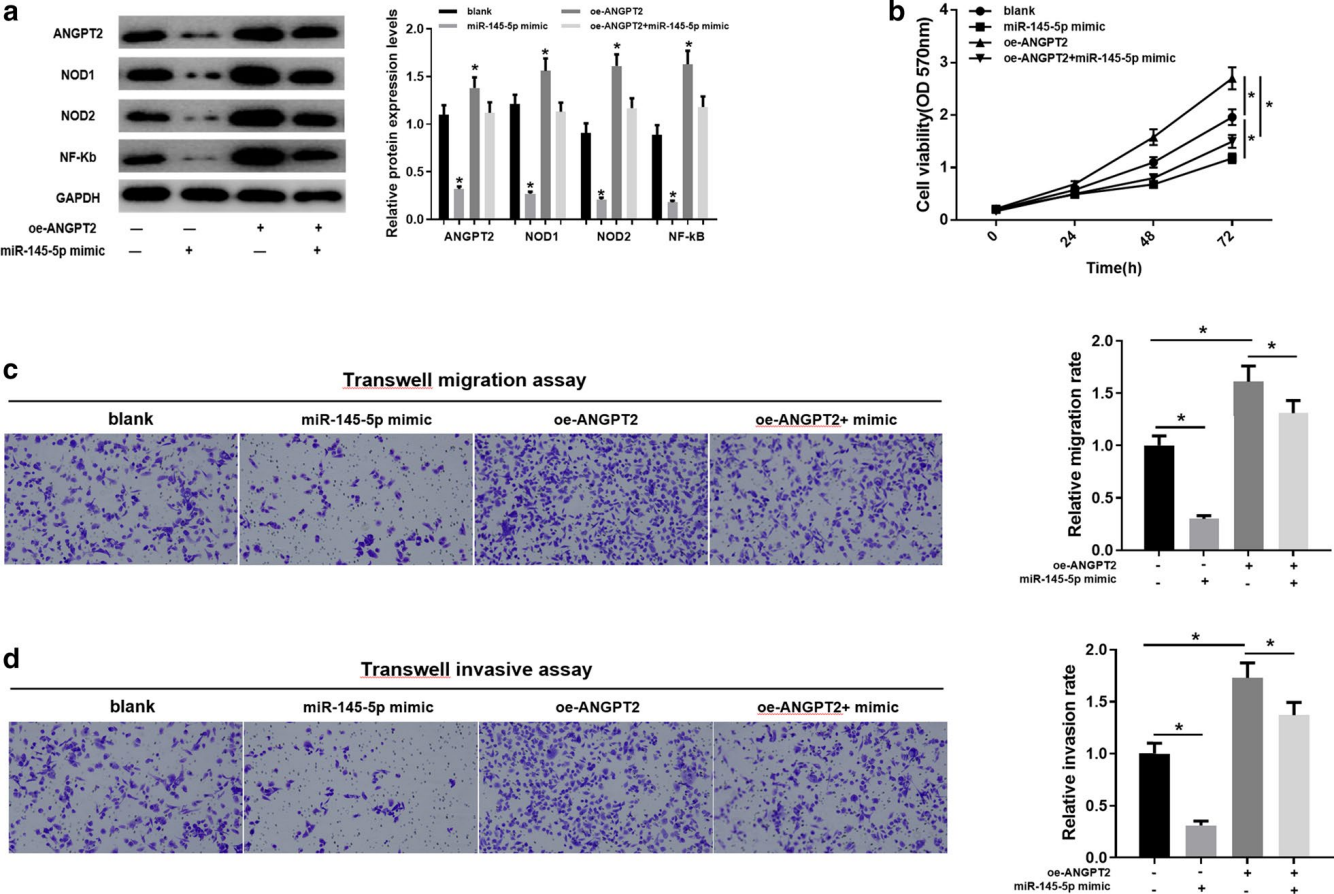
Overexpression of miR-145-5p inhibits the NOD_LIKE_RECEPTOR pathway. **a** Protein levels of ANGPT2, NOD1, NOD2 and NF-κB in the blank, miR-145-5p mimic, oe-ANGPT2 and oe-ANGPT2 + miR-145-5p mimic groups in AGS cells are detected by western blot; **b**–**d** Cell proliferation, migration and invasion of AGS cells in each group are tested by MTT, Transwell migration and invasion assays; *p < 0.05

In previous experiments, *ANGPT2* had been verified to function on the proliferation, migration and invasion of GC cells via the NOD_LIKE_RECEPTOR pathway. As *ANGPT2* had been confirmed as a target of miR-145-5p, western blot was firstly performed to investigate the regulatory relationship between miR-145-5p and the *ANGPT2*/NOD_LIKE_RECEPTOR axis. The results showed that the expression of *ANGPT2* was suppressed in the miR-145-5p mimic group with the blank group as control. In addition, in the cells with overexpression of miR-145-5p and *ANGPT2*, ANGPT2 expression was significantly lower than that in the oe-ANGPT2 group, which might be attributed to the decrease of *ANGPT2* synthesis rate caused by the targeting effect of miR-145-5p. Meanwhile, protein levels of NOD1, NOD2 and NF-κB in the NOD_LIKE_RECEPTOR pathway were detected and found to be relatively high in the groups with high expression of *ANGPT2*. When miR-145-5p was over-expressed, the protein levels of these proteins were down-regulated to approach to the normal (Fig. 5a).

Furthermore, according to the MTT assay, tumor cells with higher protein levels of NOD1, NOD2 and NF-κB had a relatively high growth rate (Fig. 5b). Transwell migration and invasion assays revealed that compared with the blank group, cell migration and invasion were remarkably inhibited in the miR-145-5p mimic group but significantly enhanced in the oe-ANGPT2 group, as shown in Fig. 5c, d. Moreover, in the oe-ANGPT2 + miR-145-5p mimic group, cell migration and invasion were significantly decreased than those in the oe-ANGPT2 group, which was in agreement with the results obtained above. In sum, miR-145-5p was considered to function on the proliferation, migration and invasion of GC cells via the *ANGPT2*/NOD_LIKE_RECEPTOR axis.

## Discussion

MiRNAs are common in eukaryotes and have been verified to be involved in the regulation of about one third human genes via genome analysis. Each miRNA has hundreds of target genes and participates in the translation or degradation of mRNA in the way of base pairing [32, 33]. Calin et al. found [34] that among 186 miRNAs, 98 miRNAs (52.5%) locate at the cancer-related genome regions or fragile sites, and Northern blot results showed that miRNAs in the deleted regions are found to be down-regulated in cancer samples. Many reports have verified that miRNAs can serve as an oncogene or a tumor suppressor gene in cancers, and most of them play an important role in the occurrence and development of GC. For instance, miR-378 inhibits the epithelial–mesenchymal transition (EMT) process, suppresses cell migration and invasion of GC by targeting *BMP2* [35]. MiR-107 functions on cell growth and metastasis of GC via the *FAT4*/PI3K_AKT axis [36]. MiR-145-5p plays a part in various cancers by pairing with its target genes, such as clear cell renal cell carcinoma [37], esophageal squamous carcinoma [38], lung cancer [39] and bladder carcinoma [40]. In GC, miR-145-5p targets *N-cadherin* and *ZEB2* to inhibit EMT, thereby inhibiting cell invasion [41]. Furthermore, studies have found that miR-145-5p is down-regulated in undifferentiated GC cells, and directly targets *KLF5*, thus affecting cell differentiation [42]. In the present study, we confirmed that miR-145-5p was down-regulated in GC cells and significantly associated with cell proliferation, migration and invasion. Therefore, it’s of great importance to further elucidate the molecular mechanism of miR-145-5p underlying the tumorigenesis and development of GC.

As previously described, angiogenesis is crucial in the occurrence and development of various malignancies. Thus, molecular treatment methods like the application of the inhibitors of angiogenesis are increasingly important [43]. In most solid tumors, the formation and expansion of blood vessels are tightly correlated with the activity of VEGFs. *ANGPT2*, a factor suppressed by VEGFs, has been studied on its activity in clinical tumor models, and its up-regulation has been considered as one of the mechanisms of acquired drug-resistance during anti-VEGFs treatment [44]. In addition, studies have found that the expression level of *ANGPT2* in serum might be a potential predictor in lung cancer staging and correlated with prognosis [45]. Meanwhile, a phase III randomized trial of AVAGAST made by Hacker, et al. revealed that *ANGPT2* can function in GC by acting as a biomarker [21]. During our differential analysis based on the TCGA-STAD dataset, *ANGPT2* was found to be a potential target of miR-145-5p and significantly up-regulated in GC. Moreover, miR-218 has been reported to inhibit the proliferation and invasion of GC cells through regulating *ANGPT2 *[29]. Lu et al. confirmed that miR-145 plays an anti-tumor role in renal cell carcinoma and it targets two oncogenes including *ANGPT2* and *NEDD9 *[31]. However, whether the regulatory relationship between miR-145 and *ANGPT2* functions on GC cells has not been reported yet. In our study, we identified that *ANGPT2* was remarkably increased in GC cells, and miR-145-5p could target *ANGPT2* mRNA to inhibit the expression of *ANGPT2*, which is consistent with previous studies.

Besides, KEGG enrichment analysis showed that high expression of *ANGPT2* was correlated with the NOD_LIKE_RECEPTOR pathway. Activated NOD1 and NOD2 receptors can be involved in the immunoreaction through the activation of NF-κB, JNK, p38, MAPK and ERK signaling pathways in the way of binding with related signal proteins like serine-threonine protein kinase RIP2, thereby promoting the secretion of various factors, such as IL-6, IL-8, IL-1β and TNF-α [46]. Previous studies suggested that estrogen receptor α regulates the Wnt/β-catenin signaling pathway via NOD-like receptors [47]. In our study, results of western blot showed that in GC cells, the protein levels of NOD1, NOD2 and NF-κB were remarkably increased but significantly decreased after silencing *ANGPT2*. Thus, we speculated that *ANGPT2* could participate in angiogenesis and the proliferation and migration of cancer cells probably through the NOD_LIKE_RECEPTOR signaling pathway.

## Conclusions

In conclusion, our study verifies that miR-145-5p affects the proliferation, migration and invasion of cancer cells via the *ANGPT2*/NOD_LIKE_RECEPTOR axis, which helps the mechanism research underlying GC cancer progression.

## Data Availability

The data used to support the findings of this study are included within the article. The data and materials in the current study are available from the corresponding author on reasonable request.
